# New Insights in Pathogenesis of Endometriosis

**DOI:** 10.3389/fmed.2022.879015

**Published:** 2022-04-28

**Authors:** Pietro G. Signorile, Rosa Viceconte, Alfonso Baldi

**Affiliations:** ^1^Italian Endometriosis Foundation, Rome, Italy; ^2^Department of Environmental, Biological and Pharmaceutical Sciences and Technologies, Università degli Studi della Campania Luigi Vanvitelli, Caserta, Italy

**Keywords:** endometriosis, fetal life, critical period, adenomyosis, coelomic metaplasia

## Abstract

Endometriosis is a gynecological disease characterized by the growth of endometrial glands and stroma outside the uterine cavity. The incidence of the disease is very high, there are currently no reliable early diagnostic tests, the therapies are only symptomatic and, consequently, the social impact of endometriosis is very important, also considering the related fertility problems. Despite this, the pathogenesis of endometriosis is still not fully defined. Retrograde menstruation and coelomic metaplasia are currently the most recognized pathogenetic hypotheses. Recent experimental evidences generated by our research group and by others have indicated an alteration of the fine-tuning of the female genital system developmental program during a critical window of time in the fetal life as the pathogenetic event prompting to the development of endometriosis later in life. Goal of this article is to present a revision of the recent literature about the different pathogenetic mechanisms proposed for endometriosis with particular emphasis on the embryologic theory. The possible clinical and pathological implications of these findings will be discussed.

## Introduction

Endometriosis is a pathological condition characterized by the presence of endometrial structures including glands and stroma, outside the uterine cavity. The growth of these endometriotic structures, due to the estrogenic hormonal input, causes a chronic inflammatory state in the pelvic level, because the endometriotic tissue, unlike the endometrial one, cannot be eliminated through menstruation at the end of the maturation process. Unfortunately, this pathology is very common affecting up to 10% of all women (1). This incidence rises to 30–50% in women also suffering from chronic pelvic pain and infertility (2, 3). As regards the localization of endometriotic implants, the most common anatomical sites are in the deep peritoneum, while the superficial peritoneum has a low prevalence (4). Moreover, the occurrence of endometriotic lesions is also noteworthy in the pouch of Douglas (4). This last specific disorder in which endometriotic lesions are present under the peritoneum, is named deep-penetrating endometriosis and is strongly linked with pelvic pain symptoms and infertility (5, 6).

Endometriosis patients commonly necessitate extensive medical and surgical treatments, with important connected costs and risks (7). Despite the fact that this disease is very common and causes significant morbidity in those who suffer from it, endometriosis is an incredibly underdiagnosed and undertreated pathology, with an irrationally long interval of 8–12 years between the beginning of the symptoms and a definitive diagnosis (8). The reason for this is the fact that the majority of the symptoms are non-specific and, at present, there are no non-invasive diagnostic tests capable of defining a diagnosis of certainty (9). In relation to this problem, our research group has recently identified some possible molecular diagnostic markers for endometriosis, and studies are underway to possibly confirm the diagnostic validity of these markers (10–12). At the present time, the definitive diagnosis of endometriosis can be attained solely through histological examination of ectopic implants obtained by invasive surgical or laparoscopic procedures (1). For all these reasons, endometriosis is a disease that has a major social and economic impact with detrimental effects upon women’s working activity, personal life, and relationships with physicians (6).

The main reason for which, to date, there are no definitive diagnostic and therapeutic paths for this disease, lies in the fact that the pathogenetic mechanism that causes it has not yet been defined with certainty (13). The purpose of this article is to analyze the most accredited theories on the pathogenesis of endometriosis with particular emphasis on the embryogenetic one. In detail, we performed a systematic review, searching on PubMed all articles dealing with endometriosis pathogenesis, using as query the words “endometriosis,” “pathogenesis,” and “review.” More than three thousand articles published between 1957 and 2021 were analyzed and almost two hundred were selected for careful reading.

## Pathogenesis of Endometriosis

Thanks to the works of Knapp and that of Nezhat, the phases in the discovery and characterization of endometriosis in the history of medicine are now very well-defined (14, 15). Although the symptoms of the disease are described from over 2000 years ago, it is only in the last century that the pathology has been clearly defined. Nevertheless, the pathogenetic mechanisms responsible of endometriosis have not been definitively elucidated. Presently, several pathogenetic theories have been proposed to elucidate the development and establishment of endometriosis. Among the most accepted theories are retrograde menstruation, hematogenous or lymphatic spread, coelomic metaplasia, and extrauterine-sourced stem cells. Recently, an embryogenetic theory with Müllerian rest induction has also been proposed (16). Table 1 summarizes the different theories on the pathogenesis of endometriosis.

**TABLE 1:** Theories proposed for the etiology of endometriosis.

Theory	Mechanism proposed
Retrograde menstruation theory	Retrograde menstruation permits implantation of endometrial glands and stroma into the peritoneal cavity (13)
Coelomic metaplasia theory	Endometriosis can develop in all celomic wall derivatives because of a metaplastic phenomenon (27)
Hematogenous/lymphatic spread theory	Dissemination of endometrial cells takes place by lymphatic or hematogenous vessels (35)
Stem cell recruitment theory	Endometrial and/or hematopoietic stem cells could differentiate into endometriotic tissue at different anatomical sites (37)
Embryogenetic theory	Persistence of residual embryonic cells of Wolffian or Müllerian ducts may develop into endometriotic lesions in response to estrogen (17)

*The most relevant references are indicated.*

### Retrograde Menstruation Theory

The most widely accepted theory for the origin of endometriosis has been for a long time that of retrograde menstruation, proposed one century ago by Sampson (17). This theory claims that at menstruation some of the flaked tissue flows retrograde through the fallopian tubes into the peritoneal cavity, causing the adhesion and growth of endometriosis structures. This mechanism considers endometriosis an auto-transplant of normal endometrial tissue in an ectopic location. It explains some the most common superficial sites of endometriosis, such as the mucosa of fallopian tubes, the subserosa of the fallopian tube, the visceral organs, the peritoneal wall and the ovarian endometriotic cysts (4) and it is supported by the fact that women with uterine outflow obstruction have a higher risk of endometriosis (18). Moreover, retrograde menstruation is a commonly described event in a very high percentage of women having blood in their pelvis at the time of menstruation (19).

On the other hand, it is indisputable that there are numerous clinical and experimental evidences that do not support the validity of this theory. First of all, the retrograde menstruation model it is not suitable for explaining the occurrence of deep endometriosis (20). In this condition, the endometriosis lesions are located deep in the organ structures of the pelvis under the peritoneum surface. For the same reason, is challenging to apply this theory to the presence of endometriosis in remote areas outside the peritoneal cavity, such as the lungs, skin, lymph nodes, and breasts (20). Moreover, it is not an acceptable pathogenetic mechanism for endometriosis described in adolescents and even in newborns (21, 22), as well as in women affected by the Mayer-Rokitansky-Küster-Hauser syndrome, a disease characterized by congenital aplasia of the uterus and the upper part of the vagina (23). Consistently, it cannot be considered a valid pathogenetic mechanism in cases of male endometriosis. This is a rare event, but well described in the scientific literature (24). Finally, well-designed observations by Redwine suggest that endometriotic tissue does not display features of an auto-transplant (25).

### Hematogenous/Lymphatic Spread Theory

The lymphatic and vascular spread theory proposes the dissemination of endometrial cells by lymphatic or hematogenous vessels. In this model, it would be necessary to assume that the menstrual endometrium containing both epithelial and stromal cells can pass into angiolymphatic circulation without disruption and that it is able to undergo extravasation from the vessels in order to be located within the muscular layers of organs. Even if the pathology of deep endometriosis is similar to cancer metastasis, these are all typical characteristics of tumor cells and, to date, there are no scientific evidences to support the idea that menstrual endometrium arising from benign endometrium is able to realize these challenging cancer-like tasks (20).

### Coelomic Metaplasia Theory

In 1942, Gruenwald described the theory of celomic metaplasia (26). This theory claims that celomic walls (peritoneal serosa or serosa-like structures) are embryologically related to Mullerian ducts and hence endometriosis can develop in all celomic wall derivatives because of a metaplastic phenomenon (27). Endocrine-disrupting chemicals may also have a role in the transformation/activation of these celomic cells, as suggested by several studies (28). This theory would explain the cases where retrograde menstrual flow is impossible.

### Stem Cell Recruitment Theory

Stem cells are undifferentiated cells that have the capacity to self-renew and to produce more differentiated daughter cells. There exist two main alternatives of the theory based on the tissue origin of the stem cells. The first one claims that stem cells arise from the uterine endometrium. The second one, instead, proposes that the origin of the stem cells is the bone marrow (20). Unrelatedly of the place of stem cell origin, hormones and other molecular factors in the tissue microenvironment, then, are supposed to contribute to all the steps necessary for the establishment of endometriosis.

Concerning the epithelial stem cells of the endometrium, they are thought to be localized in the terminal ends of the basalis glands at the endometrial/myometrial interface, and to be involved in the regeneration of the endometrial epithelium upon the estrogenic input, as it is the case for all the epithelial tissues (29). Unfortunately, researchers still not have individuated specific markers for endometrial epithelial stem cells (30). In contrast to endometrial epithelial stem cells, mesenchymal stem cells not only are normally present in the endometrium (31), but they can be readily isolated and enriched (32). Therefore, they have been intensively studied. These cells are localized in the perivascular area of the basal layer, and they are in charge for generating functionalis stroma. They can be proficiently isolated from endometrial biopsies or from shed menstrual blood using combinations of several well-defined molecular markers, such as CD146, PDGFR-B, and SUSD2 (30, 33). It is hypothesized that stromal stem cells of the endometrium, through retrograde menstruation are directed outside of the uterus, and their differentiation would be the cause endometriosis.

Some studies suggest the development of endometriosis from stem cells in the bone marrow (34). These stem cells in several experimental settings contribute to the generation of both epithelial and stromal cells, displaying a slight preference for stroma (35). According to the theory, if bone marrow stem cells are misplaced in soft tissue rather than homing back to the endometrium, endometriosis can then develop. There are several experimental evidences supporting this theory (35, 36). Nevertheless, stem cells of the bone marrow are thought to be the main font of stem cells that cause the formation of endometriosis outside of the peritoneal cavity, and they could be also the source of the rare endometriosis cases in men (37, 38).

### Embryogenetic Theory

The theory of embryonic cell remnants postulates that the persistence of residual embryonic cells of Wolffian or Müllerian ducts may develop into endometriotic lesions in response to estrogen (13). The organogenesis of the female reproductive tract from the Müllerian ducts is regulated by multifaceted spatio-temporal molecular pathways, including the anti-Müllerian hormone signaling (39). Anomalous differentiation or relocation of the Müllerian ducts in a critical period during embryogenesis could cause the spread of primordial endometrial cells in their migratory pathway across the posterior pelvic floor. These nests of cells would remain dormant until puberty, when the activation of the estrogenic hormonal input would cause their proliferation and, therefore, the onset of endometriosis with its characteristic symptoms. Furthermore, this mechanism would explain the frequent localization of endometriosis in the deep peritoneum and in the pouch of Douglas (4).

This pathogenetic mechanism was proposed by pioneer researchers of this disease in the late 19th and 20th century, but mysteriously forgotten after the affirmation of Sampson’s retrograde menstruation theory (14, 15). Further supporting the theory of the fetal origin is the observation that in adolescent patients, the cells of the endometriosis structures preserve some distinctive characteristics of fetal endometrium cells, such as ontogenic resistance to progesterone (40).

Recently, through a careful autopsy analysis, our research group has highlighted the presence of endometriotic structures in a significant number of female fetuses (41–43). It is interesting to note that these observations have been independently confirmed in subsequent work by other research groups (44). These scientific evidences clearly support the embryogenetic theory.

**FIGURE 1:**
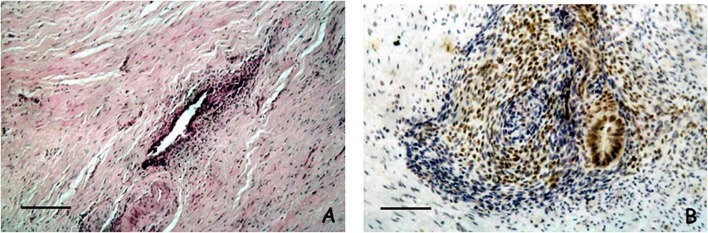
**(A)** Microscopical appearance of a case of ectopic endometrium found in the Douglas pouch. The histological section shows an endometriotic gland and a thin cuff of peri-glandular endometriotic stroma (Scale bar = 100 μ) (Hematoxylin and Eosin, original magnification × 20). **(B)** Immunohistochemical analysis of estrogen receptor in a case of ectopic endometrium found close to the posterior wall of the uterus. Both the endometriotic gland and the peri-glandular stroma display reactivity for estrogen receptor (Scale bar = 100 μ) (Immunohistochemical staining performed with the Avidin-Biotin Complex methodology, original magnification × 20).

We have analyzed by autopsy a total number of one hundred female fetuses and found ten cases of with the presence of ectopic endometrium (41–43). In detail, we carried out a careful dissection of the entire pelvic excavation, analyzing it in its entirety through serial sections. We have identified these histological structures outside the uterine cavity and they could not be ascribed to any normal histological formation. The anatomical sites of these ectopic endometrial structures were: the Douglas pouch, the recto-vaginal septum, the mesenchymal tissue close to the posterior wall of the uterus, the rectal tube at the level of muscularis propria, and the wall of the uterus. Remarkably, all of these anatomical locations are very well-known sites for endometriosis in women (4). Figures 1A,B display an example of the histological and immunohistochemical appearance of this ectopic endometrium.

We also carried out immunohistochemical studies to better define the histogenesis and molecular characteristics of these endometriotic structures. From an immunohistochemical point of view these structures were organoids with an epithelial component expressing the estrogen receptor, CA-125, and cytokeratin 7, while the stromal cells exhibited positive staining for both CD-10 and estrogen receptor. Interestingly, the fetal endometrium was also evaluated in these patients and revealed identical staining patterns. The data from the histological and immunohistochemical analysis clearly demonstrate that these structures are morphologically and from an immunophenotypic point of view attributable to ectopic endometrium located outside the uterine cavity during the earlier steps of organogenesis. To the best of our knowledge, these observations have been the first direct and systematic demonstration of the theory of embryonic cell remnants as the cause of endometriosis.

As already anticipated before, these data were subsequently confirmed in a totally independent way by another research group (44). In detail, de Jolinière et al. analyzed the reproductive organs of seven female fetuses at autopsy. In their work, they demonstrated in two out of seven fetuses, ectopic endometrial glands in the myometrium. Moreover, they found in six fetuses some ectopic endometrial glands surrounded by stroma in the uterine broad and ovarian ligaments and under the fallopian tube serosa. These ectopic endometrial glands from an immunohistochemical point of view displayed a phenotype very similar to the one described in our work with a positive staining for estrogen and progesterone receptors in the epithelial components, while the stromal counterpart presented positive staining for CD-10 and vimentin.

From a careful analysis of the scientific literature, it is possible to highlight the existence of some anecdotal scientific observations supporting our data (22). As an example, Schuster and Mackeen described a case of fetal endometriosis diagnosed as a large fetal pelvic mass at 35 weeks of gestation, and histologically confirmed to be a cystic endometriosis of the left ovary (45). Moreover, the existence of residues of Mullerian tissue dislocated during the organogenesis has been proposed by several research groups (46, 47). It is also interesting to note the fact that numerous studies have shown a close correlation between uterine malformations or Mullerian duct anomalies and endometriosis (48–50). Finally, using a genomic approach, our research group has recently demonstrated that the transcriptome of endometriotic tissue differently expresses numerous genes involved in embryogenesis with respect to endometrial tissue and that this different expression is independent of the phase of the hormonal cycle (51).

From all these observations it is possible to hypothesize a pathogenetic mechanism based on an alteration of the phenomena of embryogenesis of the uterus which would occur during a critical period of uterine morphogenesis and which would be responsible for the displacement of nests of endometrial tissue outside the uterine cavity. The exact genetic end epigenetic factors responsible of this alteration of the fine tuning of the female genital structures, that causes disruption of some organizational events associated with development of the normal neonatal uterine wall, are actually unidentified. It is very well known that estrogen is the most important hormone involved in the embryogenesis of the female genital system. Therefore, it is possible to hypothesize that an altered estrogenic hormone input could behave as a trigger, acting on a favorable genetic background, to elicit the formation of ectopic endometrium nests during embryogenesis. In favor of this hypothesis, there are numerous scientific evidences that link alterations of the female genital system and also endometriosis to exposure *in utero* to endocrine disruptors, substances capable of mimicking the action of the hormone estrogen (52–55).

## Conclusion

There is no single theory that explains all of the different clinical presentations and pathological features in endometriosis. Nevertheless, it is possible that superficial endometriosis, deep endometriosis, and ovarian endometriotic cysts develop *via* different mechanisms, and they invoke different or partially overlapping theories. At present it seems certain that the thesis promulgated in 1921 of retrograde menstruation has been set aside (17). On the other hand, the theory of the alteration of the fine tuning of the organogenesis of the female genital system due to a disturbing action of xenotoxicants or/and xenoestrogens on endometrial cells during embryonic development seems to find more and more validation. All this is further confirmed by the finding that these substances have also caused other alterations in numerous other tissues and by the impressive number of published studies concerning xenobiotics and diseases (56). It must also be underlined the fact that, unlike cancer, in which the epithelial cell is the target of the study, in endometriosis the relationship between epithelial cells and endometrium-like stromal components is the key point to determine the clonal development of endometriosis. The interplay between these two components must be clarified in several models to account for the cause of endometriosis.

In particular, it will be very important to clarify both the mechanisms that determine the enzymatic, protein and molecular changes of the epithelial and stromal endometriotic cells, and to fully understand these changes in order to be able to give a certain model of the origin of the disease and to be able to develop effective endometriosis management and therapy.

## Author Contributions

PS and AB were responsible for conceptualization, methodology, formal analysis, writing, and original draft preparation. RV was responsible for review and editing. PS was responsible for funding acquisition. All authors contributed to the article and approved the submitted version.

## Conflict of Interest

The authors declare that the research was conducted in the absence of any commercial or financial relationships that could be construed as a potential conflict of interest.

## Publisher’s Note

All claims expressed in this article are solely those of the authors and do not necessarily represent those of their affiliated organizations, or those of the publisher, the editors and the reviewers. Any product that may be evaluated in this article, or claim that may be made by its manufacturer, is not guaranteed or endorsed by the publisher.
